# Reversible autonomic dysfunction during antiviral treatment in patients with chronic hepatitis C virus infection: Anti-HCV therapy and autonomic function

**Published:** 2011-02-01

**Authors:** Janos Osztovits, Evelin Horvath, Judit Tax, Levente Csihi, Tamas Horvath, Levente Littvay, Tamas Toth, Margit Abonyi, Peter L. Lakatos, Mark Kollai, Janos Feher, Ferenc Szalay, Hubert E. Blum

**Affiliations:** 1 First Department of Internal Medicine, Semmelweis University, Budapest, Hungary; 2 Institute of Human Physiology and Clinical Experimental Research, Semmelweis University, Budapest, Hungary; 3 Central European University, Budapest, Hungary; 4 Second Department of Internal Medicine, Semmelweis University, Budapest, Hungary; 5 Second Department of Medicine, University of Freiburg, Freiburg, Germany

**Keywords:** Autonomic dysfunction, Antiviral treatment, Hepatitis C virus

## Abstract

**Background:**

The first clinical sign of chronic hepatitis C virus (HCV) infection can be one of the various extrahepatic manifestations. During antiviral treatment, symptoms of HCV-associated neuropathies usually improve, but can also worsen and lead to discontinuation of anti-HCV therapy. Recently, we have reported autonomic dysfunction in patients with HCV infection.

**Objectives:**

In the present prospective study, we analyzed the changes of autonomic function during anti-HCV treatment.

**Patients and Methods:**

Cardiovagal autonomic function was assessed in 22 HCV RNA-positive, treatment-naive patients by determining heart rate variability (HRV) and baroreflex sensitivity (BRS), at the beginning of treatment and 12, 24 and 48 weeks of antiviral therapy. interferon alfa-2 and ribavirin were given according to the guidelines.

**Results:**

Both HRV and BRS time and frequency domain indices decreased after 12 weeks of therapy compared to the pre-treatment values; then the mean±SD values increased significantly by week 24 and continued to improve by week 48 of therapy-253.0±156.1 ms before therapy vs 111.6±81.9 at week 12, and 183.4±169.6 at week 24 vs 211.6±149.1 ms at week 48 for low-frequency HRV index; p<0.05 for all comparisons). These changes were independent from the presence of cryoglobulins and from virologic response.

**Conclusions:**

The first rise followed by reversible autonomic dysfunction during antiviral therapy may be caused by the immunomodulatory actions of interferon alfa-2.

## Background 

Chronic hepatitis C virus (HCV) infection is a common cause of chronic liver disease and liver cirrhosis; it is the main cause of hepatocellular carcinoma [1]. The first clinical sign of HCV infection can be an extrahepatic manifestation, which develops in more than 50% of patients during the course of the disease, even in the absence of hepatic symptoms [2][3]. Among HCV-associated extrahepatic manifestations, neurological complications are common, including impairment of both peripheral and central nervous system, e.g., peripheral motor and sensory dysfunctions, transverse myelopathy, cognitive impairment, fatigue, anxiety, depression, and impaired quality of life [4][5][6]. Autonomic function can be assessed by reproducible non-invasive methods. Parasympathic activity, basal vagal outflow can be determined by measuring various indices of heart rate variability (HRV), and autonomic integrative function can be estimated by measuring baroreflex sensitivity (BRS) indices [7][8]. Depressed HRV and BRS have been established as independent predictors of cardiovascular morbidity and mortality [9][10]. In addition, autonomic nerve dysfunction is well described in chronic alcoholic and non-alcoholic liver diseases, including primary biliary cirrhosis and chronic hepatitis B virus infection. Moreover, autonomic neuropathy in chronic liver disease is associated with a five-fold increase in mortality within four years, independent from the severity of the liver disease [11]. Autonomic dysfunction can show improvement as well, in patients with end-stage after liver disease after transplantation [12]. In our previous study, we have first described impaired cardiovagal autonomic function in patients with chronic HCV infection [13]. The response of extrahepatic manifestations to the current standard of antiviral therapy is mostly favorable [14]. However, no response, worsening and even the development of central and peripheral neurotoxicity have been reported, as well, including sensory and motor neuropathies, depression, irritability and anxiety, leading to temporary or permanent discontinuation of antiviral therapy [15][16][17][18]. To date, it is not known how antiviral therapy affects autonomic nervous function in patients with chronic HCV infection.In the present study, we longitudinally assessed cardiovagal autonomic function by determining HRV and BRS indices in patients with chronic HCV infection during antiviral therapy in parallel with markers of liver cell damage (serum aminotransferases), liver synthetic capacity (serum albumin), glucose metabolism, and serum cryoglobulins.

## Patients and Methods

### Subjects

We followed 22 patients with chronic HCV infection during antiviral therapy, and performed autonomic function and laboratory examinations one day before therapy, and on 12, 24 and 48 weeks of the treatment. Patients were recruited from three outpatient liver clinics in Budapest, Hungary, between January 2006 and December 2007. Inclusion criteria were 1) HCV RNA positivity; 2) no previous antiviral treatment; 3) no known liver disease in history other than chronic HCV infection; 4) no HIV or hepatitis B virus (HBV) co-infection; 5) no alcohol or drug abuse; 6) no histological, laboratory or clinical evidence of liver cirrhosis; 7) no uncontrolled psychiatric condition, pregnancy or lactation; 8) no disease that might affect autonomic nerve system, such as diabetes, hypertension, heart failure, ischemic heart disease, end-stage renal disease, stroke, Parkinson's disease, or AIDS; 9) no treatment influencing autonomic nervous system such as ß-blockers, muscarinic receptor blockers; and 10) having a sinus rhythm. For antiviral therapy, a white blood cell count <3,000/mm3, absolute neutrophil count <1,500/mm3, and platelet count <80,000/mm3 as well as uncertainty about effective contraception during therapy were contraindications. All individuals gave written informed consent to participate in the study. This study was approved by the Ethics Committee of the Semmelweis University, Budapest, Hungary.

### Study design

Patients were treated with pegylated interferon (PEG-IFN) α-2a (40 kd, 180 μg) or PEG-IFN α-2b (12 kd, 1.5 µg/kg) subcutaneously once per week and ribavirin (1000 mg if wt<75 kg and 1200 mg if wt>75 kg) per os daily. Duration of therapy was considered individually according to the guidelines. Treatment responses were characterized by the results of HCV RNA testing. Early virologic response was defined as a decrease in serum HCV RNA concentration to <50 IU/mL or a decrease of at least 2 log units from baseline viral load 12 weeks of therapy. SVR was defined as negativity for HCV RNA in serum by a PCR test at the end of the treatment and six months later. Autonomic function and laboratory examinations were done one day before the therapy, then on 12, 24 and 48 weeks of the treatment, depending on the individual duration of antiviral therapy.

### Laboratory analyses

Patients underwent laboratory examination one day before autonomic function tests. Routine laboratory tests included AST, ALT, albumin and blood glucose levels. HCV RNA was quantified by real-time PCR (COBAS TaqMan, Roche Diagnostics, Meylan, France). For the detection of mixed cryoglobulins, blood samples were kept at 37 °C until complete coagulation and analyzed by standard methods.

### Cardiovagal autonomic function

Blood pressure, heart rate and respiration

Radial artery pressure was monitored continuously with an automated tonometric device (Colin CBM-7000; Colin Corp., Komaki City, Japan) for determination of BRS indices. Systolic (SBP) and diastolic blood pressure (DBP), measured on the brachial artery by an automatic microphonic sphygmomanometer built into the Colin device, were used to calibrate the radial pressure pulse. RR intervals (RRIs) were measured from R-wave threshold crossings on continuously recorded electrocardiogram (ECG). Respiration was recorded with an inductive system (Respitrace System; Ambulatory Monitoring, Ardsley, NY, USA). To improve the reliability of measurements, breathing rate was paced at 0.25 Hz [19].

### Baroreflex sensitivity (BRS)

The coupling between spontaneous fluctuations in heart rate and SBP was determined by the sequence method and by spectral analysis. The software used (WinCPRS program; Absolute Aliens Oy, Turku, Finland) detected the ECG R wave, computed RRI and radial artery SBP time series and identified spontaneously occurring sequences in which SBP and RRI concurrently increased and decreased over three or more consecutive beats (BRSseq). The minimal accepted change was 1 mm Hg for SBP and 5 ms for RRI. Only sequences with a correlation coefficient >0.85 were considered. To determine spectral indices, the signals were interpolated, resampled and their power spectra were determined using fast Fourier transformation-based methods. The LFgain (LF [Low-Frequency] transfer function gain) was determined, which expresses RRI and SBP cross-spectral magnitude in the frequency range of 0.05-0.15 Hz, where coherence is >0.5.

### Heart rate variability (HRV)

Time and frequency domain measurements of HRV from 10-min recordings of RRIs were calculated using the WinCPRS program. Non-sinus beats were semi-automatically removed and corrected using interpolation of preceding beats. The following parameters were determined: the standard deviation of the RRI (termed NNSD), the root mean square of successive differences (termed RMSSD), the percentage of successive RRIs that differed by >50 ms (termed pNN50), as well as low-frequency (0.05-0.15 Hz) and high-frequency (0.15-0.4 Hz) power of RRI variability (termed LF and HF, respectively).

### Examination of cardiovagal autonomic function

Patients were studied in the early afternoon under standardized conditions, in a quiet room at a comfortable temperature. All individuals fasted at least two hours before testing and were asked to refrain from strenuous exercise or drinking alcohol or caffeinated beverages for 24 hours before this study. Subjects were equipped with the appropriate devices, and then rested in the supine position for approximately 15 minutes until baseline conditions for heart rate and mean blood pressure were reached. Subjects were then asked to synchronize their respiratory rate with a metronome beating at 0.25 Hz. RR intervals and radial artery pressure were recorded continuously for 10 minutes to determine spontaneous baroreflex indices.

### Data analysis

Blood pressure and ECG recordings were digitized and analyzed using the WinCPRS program using a sampling rate of 500 Hz. Data were expressed as means±SD. To assess the changes over time in variables of interest, repeated variance analysis was used with post hoc Tukey's test. To ensure compliance with the assumptions of repeated measures ANOVA requiring equal interval measures, two independent analyses were conducted: one with values before and at 12 and 24 weeks of treatment; and another with values before and at 24 and 48 weeks of treatment. To preserve space, results of these two analyses were combined for presentation in Table 1. To evaluate the effect of potential determinants (age, body mass index [BMI], serum ALT, albumin, glucose, cryoglobulinemia, HCV RNA, and response to therapy) on autonomic (HRV and BRS) indices, we performed a multivariate analysis using full information maximum likelihood regression including all selected participants and correcting for attrition. To correct for any violations of residual normality, robust standard errors were used. Serum HCV RNA level was log transformed to correct its highly skewed distribution. The null hypothesis of no relationship was rejected at p<0.05. Analyses were performed using Mplus version 5.2 (Muthen and Muthen, Los Angeles, CA, USA).

**Table 1:** Laboratory data and autonomic function indices in patients with chronic hepatitis C during antiviral treatment

**Parameter**	**Treatment^e^**			
	**Before**	**Week 12**	**Week 24**	**Week 48**
**No **(male)	22 (11)	22 (11)	21 (11)	19 (10)
**AST a**(IU/L) normal: 12-38	92.1 ± 56.2	45.7 ± 27.7 b	39.5 ± 26.7 b	42.16 ± 23.8 b
**ALT a** (IU/L) normal: 7-41	113.3 ± 71.5	37.8 ± 26.1 b	33.2 ± 27.8 b	33.7 ± 21.8 b
**Albumin **(g/L) normal: 40-50	45.8 ± 7.1	43.8 ± 4.3	45.6 ± 3.7	43.9 ± 4.3
**Glucose **(mmol/L) normal: 4.2-6.1	5.1 ± 0.5	5.1 ± 0.7	5.1 ± 0.6	5.0 ± 0.5
**Cryoglobulinemia**(%)	8 (35%)	5 (22%)	2 (8%)	1 (5%)
**Heart rate **(beats/min)	73.1 ± 6.0	76.2 ± 7.5	73.4 ± 8.1	75.2 ± 9.2
**NNSD a**(ms)	36.8 ± 11.9	27.5 ± 10.8 c	31.3 ± 16.1	34.1 ± 11.3
**RMSSD a**(ms)	28.0 ± 14.7	19.9 ± 13.5	29.9 ± 23.8	30.2 ± 17.3
**pNN50 a**(%)	5.5 ± 6.2	1.4 ± 2.3 c	3.4 ± 4.4 d	4.4 ± 4.9
**LF a**(ms2)	253.0 ± 156.1	111.6 ± 81.9 c	183.4 ± 169.6 d	211.6 ± 149.1
**HF a**(ms2)	230.1 ± 165.1	105.0 ± 97.9 c	254.5 ± 333.4 d	251.8 ± 290.4
**BRSseq a**(ms/mmHg)	8.1 ± 3.9	5.6 ± 2.0 c	8.3 ± 4.1 d	8.8 ± 2.9
**LFgain a**(ms/mmHg)	6.8 ± 3.6	5.0 ± 2.2 c	5.9 ± 2.6 d	6.7 ± 2.4

^a^ AST: Aspartate aminotransferase; ALT: Alanine aminotransferase; NNSD: SD of RR intervals; RMSSD: Root mean square of successive RR interval differences; pNN50: Percentage of RR intervals that differ >50 ms; LF: Low-frequency (0.05-0.15 Hz) power of RR interval variability; HF: High-frequency (0.15-0.4 Hz) power of RR interval variability; BRSseq: Baroreflex sensitivity sequence index; LFgain: Cross-spectral transfer gain in the low-frequency range.

^b^ Significantly different (p < 0.001) from "Before treatment" value

^c^ Significantly different (p < 0.01) from "Before treatment" value

^d^ Significantly different (p < 0.05) from "Treatment week 12" value

^e^ Values are given as means ± SD. Normal laboratory values are given according to reference No [38].

## Results

Twenty-two patients were eligible and started antiviral treatment. The participants had a mean±SD age of 44.9±10.1 (range: 26-62) years, and BMI of 26.1±5.4 (range: 19.8-40.4) kg/m2. The mean serum HCV RNA level was 1,900,000 (range: 5,800-10,700,000) IU/mL. Genotyping revealed HCV subtype 1b in 21 (95%) of 22 patients, and subtype 1a in one (5%). Mixed cryoglobulinemia was present in eight (35%) of 22 patients before treatment. Study and treatment protocol was discontinued in one patient in week 14 because of severe fatigue. One patient died of traffic accident in week 31, and another patient refused to participate in the study examinations in week 48. Of 22 patients, 15 (68%) showed an early virological response at week 4 or 12; 11 (58%) of 19 patients achieved a sustained virological response (SVR). The patients' laboratory data and cardiovagal autonomic indices before and during antiviral treatment are shown in Table 1. The initially elevated AST/ALT levels were nearly normalized by week 12 of therapy and remained normal throughout weeks 24 and 48 (p < 0.001). Glucose or albumin levels remained unchanged before and during the therapy. Both heart rate variability sequence indices (NNSD, pNN50), frequency-domain indices (LF and HF), and baroreflex sensitivity indices (BRSseq, LFgain) were significantly decreased by week 12 (p < 0.01), and then increased by week 24 (p < 0.05) to reach pre-treatment levels by week 48 of antiviral therapy. Multivariate analyses did not identify a significant correlation between any of the anthropometric or laboratory variables studied (age, BMI, cryoglobulinemia, HCV RNA level, ALT, albumin, glucose) and changes of autonomic function indices during antiviral treatment. Response to therapy was also not associated with the changes of autonomic function.

## Discussion

Following our previous study of autonomic dysfunction in patients with chronic hepatitis C [13], we investigated the changes of autonomic function in patients with chronic HCV infection during antiviral therapy. Interestingly, a significant decrease in autonomic function occurred by week 12 of the treatment to be followed by a significant improvement by week 24 and a return to pre-treatment values by week 48 of antiviral therapy. Neurological symptoms in patients with chronic hepatitis C usually improve during antiviral therapy. However, exacerbations or even the first onset of neurological manifestations have also been observed during antiviral treatment. Ribavirin has no known neurological side effects; neither ribavirin monotherapy trials [20][21] nor randomized trials comparing the combination of IFN-α and ribavirin with IFN-ß monotherapy showed significant neurological complications [22][23]. A direct neurotoxic effect of IFN-α in our study is unlikely because autonomic dysfunction improved after three months despite the continuation of therapy. However, IFN-α may induce vasculitis with or without cryoglobulinemia, and can increase preexisting ischemia, leading to purpura, skin ulcerations, arthritis or ischemic polyneuropathy [24][25]. None of these manifestations was observed in our patients with chronic HCV infection during antiviral therapy. Presence of cryoglobulins was not associated with the changes of autonomic function. Further manifestations related to neuropathies in HCV infection may be the HCV and IFN-α treatment-related insulin resistance [26][27]. Diabetic peripheral and autonomic neuropathies are well known complications of persistent hyperglycemia [28]. Similarly, impaired cardiovagal autonomic indices are early, subclinical signs of glucose neurotoxicity in patients with diabetes [29]. In our study, patients with diabetes were excluded and fasting glucose levels did not change during antiviral therapy. Therefore, an impaired carbohydrate metabolism does not underlie the changes of autonomic dysfunction in our patients. However, the determination of the fasting glucose level only is a limitation of our study, as this method is insufficient to assess insulin resistance and glucose metabolism and possible effects of HCV-related insulin resistance on the development of cardiovagal autonomic dysfunction. Therefore, in further studies more sophisticated techniques (glucose clamp technique, homeostasis model assessment score) should be used. The immunomodulatory effect of IFN-α suggests that the immune status correlates with autonomic dysfunction. Cohort studies in HIV-positive and AIDS patients indicate that autonomic dysfunction parallels the HIV disease progression-i.e., heart rate variability indices correlate with the CD4 cell count [30][31]. The degree of autonomic dysfunction remains mild and subclinical in patients with HIV infection/AIDS in all stages of the disease, just as it was observed in our patients with chronic HCV infection. In addition, abnormal heart rate and baroreflex responses have been documented in patients with multiple sclerosis (MS) [32]. In a 24-month follow-up of 26 patients with clinically active remitting-relapsing MS, autonomic dysfunction assessed by heart rate responses correlated with the clinical activity of MS and disease progression [33]. These results are consistent with data from molecular and immunological analyses that addressed links between dysfunction of the immune regulation and the autonomic nervous system. These studies suggest that the balance between sympathic/parasympathic system plays a major role as an integrative interface between the brain and the immune system [34][35]. Consistent with this hypothesis, IFN-α in human brain physiologically affects not only the immune but also the central nervous system [36][37]. These findings suggest that the autonomic dysfunction in our patients with chronic HCV infection may be associated with changes of the immune status and IFN-α modulated immune responses. Further studies should address these interactions. In summary, the increase of cardiovagal autonomic dysfunction during the first 12 weeks of antiviral therapy is no reason to modify the treatment strategy in patients with chronic HCV infection because autonomic dysfunction remains mild and subclinical, and improves during continued antiviral therapy.
